# Community-based forensic psychiatric care for internees as a way to reduce the number of internees in prisons

**DOI:** 10.1192/j.eurpsy.2026.10232

**Published:** 2026-07-31

**Authors:** T. Marquant, K. Goethals

**Affiliations:** 1 Forensic Psychiatry, Antwerp University; 2 Forensic Psychiatry, Antwerp Universty; 3 University Forensic Centre, Antwerp, Belgium

## Abstract

**Introduction:**

Belgium’s prisons face overcrowding, with many mentally ill offenders (internees) held while awaiting placement in forensic psychiatric hospitals. Long waits and strict criteria delay care for months or years. Some are reincarcerated due to non-compliance or substance use. Psychiatric care in prison is minimal, making prolonged detention harmful. Forensic psychiatric care is mainly residential; community-based services are underdeveloped. Yet, such care can reduce hospital stays, reincarceration, and allow direct prison admissions. Other countries, like the U.S., have advanced models such as Forensic Assertive Community Treatment (FACT), backed by research. Belgium could benefit from implementing FACT alongside residential services to build a full care continuum, shorten wait times, and improve outcomes.

**Objectives:**

This study explores FACT as a community-based addition to Belgium’s residential forensic psychiatric care.

**Methods:**

The research involved four steps: We defined FACT by combining ACT elements linked to positive outcomes with forensic rehabilitation models (RNR and GLM). We then studied a FACT team in Leuven over 33 months (n=70) using a cross-sectional, controlled, retrospective design. We then focussed on substance use disorders due to their impact on outcomes, and reviewing related literature. We then designed a pilot project to reduce the impact of substance use on community tenure. In a prospective study, 24 patients were followed for one year using START (risk) and MATE (substance use). A 5-day intensive cognitive behavioural treatment was offered as an alternative to admission during relapse.

**Results:**

Six ACT elements were linked to positive outcomes: home-based treatment, embedded psychiatrist, integrated substance use and vocational therapy, small caseloads, and 24/7 service. Two forensic elements were added: hybrid professional stance and good judicial communication. FACT patients were over 6 times less likely to be rearrested and 14 times less likely to be reincarcerated, with higher community tenure. However, short admissions were frequent, mostly due to substance use. Substance use disorders were key in deciding whether patients could enter FACT directly from prison or via residential care. In the pilot, patients using the 5-day alternative postponed admission and maintained good community tenure. Risk assessments decreased, and MATE scores remained stable except for increased physical complaints.

**Conclusions:**

FACT is a safe and effective way to add community-based care to Belgium’s residential forensic psychiatric system. Its integration could reduce prison time and prevent reincarceration. Substance use disorders are critical in determining the best entry point into the care continuum to ensure safety and effective treatment.

**Disclosure of Interest:**

None Declared

